# Patient-orientated longitudinal study of multiple sclerosis in south west England (The South West Impact of Multiple Sclerosis Project, SWIMS) 1: protocol and baseline characteristics of cohort

**DOI:** 10.1186/1471-2377-10-88

**Published:** 2010-10-07

**Authors:** John P Zajicek, Wendy M Ingram, Jane Vickery, Siobhan Creanor, Dave E Wright, Jeremy C Hobart

**Affiliations:** 1 Clinical Neurology Research Group, Peninsula College of Medicine and Dentistry, University of Plymouth, UK; 2 Department of Medical Statistics, University of Plymouth, UK

## Abstract

**Background:**

There is a need for greater understanding of the impact of multiple sclerosis (MS) from the perspective of individuals with the condition. The South West Impact of MS Project (SWIMS) has been designed to improve understanding of disease impact using a patient-centred approach. The purpose is to (1) develop improved measurement instruments for clinical trials, (2) evaluate longitudinal performance of a variety of patient-reported outcome measures, (3) develop prognostic predictors for use in individualising drug treatment for patients, particularly early on in the disease course.

**Methods:**

This is a patient-centred, prospective, longitudinal study of multiple sclerosis and clinically isolated syndrome (CIS) in south west England. The study area comprises two counties with a population of approximately 1.7 million and an estimated 1,800 cases of MS. Self-completion questionnaires are administered to participants every six months (for people with MS) or 12 months (CIS). Here we present descriptive statistics of the baseline data provided by 967 participants with MS.

**Results:**

Seventy-five percent of those approached consented to participate. The male:female ratio was 1.00:3.01 (n = 967). Average (standard deviation) age at time of entry to SWIMS was 51.6 (11.5) years (n = 961) and median (interquartile range) time since first symptom was 13.3 (6.8 to 24.5) years (n = 934). Fatigue was the most commonly reported symptom, with 80% of participants experiencing fatigue at baseline. Although medication use for symptom control was common, there was little evidence of effectiveness, particularly for fatigue. Nineteen percent of participants were unable to classify their subtype of MS. When patient-reported subtype was compared to neurologist assessment for a sample of participants (n = 396), agreement in disease sub-type was achieved in 63% of cases. There were 836 relapses, reported by 931 participants, in the twelve months prior to baseline. Twenty-three percent of the relapsing-remitting group and 12% of the total sample were receiving disease-modifying therapy at baseline.

**Conclusions:**

Demographics of this sample were similar to published data for the UK. Overall, the results broadly reflect clinical experience in confirming high symptom prevalence, with relatively little complete symptom relief. Participants often had difficulty in defining MS relapses and their own MS type.

## Background

The South-West Impact of Multiple Sclerosis study (SWIMS) is a longitudinal cohort study from the perspective of people with MS. This paper outlines the rationale, methods and baseline results from the SWIMS cohort.

Multiple sclerosis (MS) is a complex and unpredictable disease with potential considerable impact on daily living, both for people with the condition and their carers. In order to develop and test new treatments in the context of clinical trials, there is a need for greater understanding of its impact, particularly from the perspective of individuals with the condition. Although people with MS have identified research into symptom relief as a high priority, there are relatively few effective symptomatic treatments, and many clinical trials of symptomatic treatments have been disappointingly negative. One reason for this may be that current methods for measuring disease impact are inadequate, and the relationship between symptoms, impairment and disability is not fully understood. In addition, measurement instruments often demonstrate limitations. For example, although fatigue is one of the most commonly reported symptoms in people with MS, and there are several scales purporting to measure the concept of fatigue, in clinical studies results using different fatigue scales may not strongly correlate with each other [1]. Given such limitations of the available measurement instruments it may not be surprising that few significant treatment effects are identified in clinical trials.

When attempting to evaluate disease impact, changes have generally been defined as objectively as possible, usually from the perspective of the neurologist, using clinical signs derived from neurological examination. There are several problems associated with this approach, including subjective interpretation of signs in a complex condition, as well as difficulties in detecting change over time. Another issue illustrating disease complexity lies in the way in which relapses are measured. Most clinical trials define relapses in terms of persistence of symptoms and signs, usually supported by changes on neurological examination. Yet we know that the frequency of changes on cranial magnetic resonance imaging (MRI) greatly exceeds the number of relapses supported by changes on neurological examination, and patients often report greater variability in their condition than can usually be confirmed by signs on neurological examination [2,3]. There is very little available information about relapses from the patient perspective, and even less data on the reliability of such information.

In trying to measure the impact of MS, to understand the natural history of MS, and establish the effectiveness of disease-modifying treatments, scales such as the Expanded Disability Status Scale (EDSS) [4] have become established as the most widely used "objective" means of measuring disease course. The EDSS is implemented by neurologists and has many accepted limitations, yet virtually all natural history studies have used the EDSS. A lack of responsiveness, amongst other limitations of the EDSS, means that clinical trials involving progressive MS patients are usually three years in duration, which can lead to a risk of patient drop-out. This may result in reduced statistical power to obtain an answer to the research question, such as occurred in the recent PrOMISe study [5]. In response to the recognised limitations of the EDSS, other instruments such as the MS Functional Composite [6] and the patient-reported MS Impact Scale-29 [7] have been developed and recommended for incorporation into clinical trials. Responsive patient-orientated rating scales have the potential to provide data which are sensitive to changes in disease. Such scales could therefore be used to model disease progression, in part to inform clinical trial methodology and also to provide relevant prognostic information for people early in the disease course. A more refined approach to providing prognostic information, from data derived over shorter periods, may enable patients and health care professionals to make better-informed decisions with regard to risk and benefit of emerging treatments. However, there are very little longitudinal data available based on these newer instruments, thus little data on which to model disease progression and to use in performing power calculations for clinical trials. Therefore there remains a need to evaluate patient-reported rating scale performance over time.

Although there have been studies evaluating prevalence of certain common symptoms such as pain, fatigue and tremor, with limited investigation of longitudinal change over time [8-10], there has been little systematic evaluation of symptoms and how they change over time, and few studies of the relationship between prevalent symptoms and other aspects of disease impact. There remains a need to understand the impact of symptoms on disease progression.

SWIMS therefore has three major aims: 1) to facilitate a better understanding of disease impact from the patient perspective, in order to develop improved measurement instruments for clinical trials and clinical practice, 2) to evaluate longitudinal performance of a variety of commonly used, patient-reported outcome measures, 3) to collect, analyse, and model patient-orientated longitudinal data in order to evaluate prognostic predictors and facilitate more individualised treatment in future patients, particularly early in the disease course.

Here we present summaries of the baseline data provided by the first 967 participants to consent to SWIMS.

## Methods

### Study Area

The SWIMS cohort is drawn from Devon and Cornwall - two counties which form a sea-bordered peninsula in south west England, covering 10,270 km^2 ^in area. The study area is situated at latitude 50.78, longitude -3.00, at the most easterly point and latitude 50.12, longitude -5.53, at the most westerly point.

The population of approximately 1.67 million people [11] is served by five acute hospitals and three neurology centres, each with its own rehabilitation centre. Devon and Cornwall are ideally suited to this type of longitudinal cohort study as the population is relatively stable and migration rates are low[12,13].

In our prevalence study of MS in an area within the study region, we found a rate of 118 per 100,000, in a population of 341,796[5]. Based on this prevalence, we estimate the number of cases of MS in the SWIMS study region to be approximately 1,800[6].

### Eligibility criteria

Inclusion criteria: clinical diagnosis of MS by either McDonald or Poser criteria, or diagnosis of clinically isolated syndrome (CIS), aged 18 years and over, and resident in Devon or Cornwall. Exclusion criterion: severe cognitive impairment such that the patient is unable to provide informed consent.

### Recruitment

The study was approved by the Cornwall and Plymouth Research Ethics Committee and the South Devon Research Ethics Committee, and adheres to the Data Protection Act 1998. SWIMS commenced in August 2004, and continues to recruit new participants. Follow-up is expected to continue until 2019.

Patients with established MS or CIS are invited to take part by one of the following routes:

1) Patients attending neurology outpatient clinics are approached by their neurology team.

2) Patients identified from review of hospital case notes are approached by their neurologist.

3) A survey of General Practitioners within Devon and Cornwall was conducted in which GPs were asked to approach eligible patients known to them who had not previously been approached by other routes.

4) Patient self-referral, in response to public awareness campaigns involving the Multiple Sclerosis Society and local media, or via information on the SWIMS website.

Informed consent is obtained to retain the data provided and to review previous medical records in order to verify diagnosis.

### Data collection

Consented MS patients are asked to complete questionnaire booklets twice per year. The full details of the questionnaire can be found at http://www.pms.ac.uk/cnrg/swims, but briefly data collection includes: type of MS (with examples of MS sub-types represented graphically in order to assist in classification, similar to Bamer *et al *and Lublin and Reingold [14,15]), relapses, symptoms, medication, investigations, contact with health and social care professionals, and whether the participant feels that he/she has deteriorated in the previous six months. The questionnaire booklet contains the following definition of a relapse: "a worsening of existing neurological symptoms which lasts for at least 48 hours, or the appearance of a new neurological symptom which lasts for at least 48 hours". A range of validated questionnaires is included (Table 1). In addition, the baseline questionnaire includes items concerning the onset of MS, e.g. date of diagnosis.

**Table 1:** Validated instruments included in questionnaire booklets

	MS Baseline Questionnaire	MS 6-Monthly Follow-Up Questionnaire Version A*	MS 6-Monthly Follow-Up Questionnaire Version B*	CIS Baseline Questionnaire	CIS 12-Monthly Follow-Up Questionnaire
EuroQol [27]	✓	✓		✓	✓

Fatigue Severity Scale [28]	✓	✓			

Functional Assessment of MS (modified 44-items scale) [29]	✓		✓		

General Health Questionnaire-30 [30]	✓	✓		✓	✓

Medical Outcomes Study Short Form 36-Item Health Study (version 2) [31]	✓		✓	✓	✓

MS Disease Impact Scale-29 (version 2) [7]	✓	✓			

MS Neuropsychological Screening Questionnaire [32]	✓		✓		

MS Walking Scale (version 2) [33]	✓	✓			

Postal Barthel Index [34]	✓		✓		

*To minimise the risk of overburdening participants, participants are randomly allocated to receive initially either Version A or Version B. Participants then receive alternate versions every six months.

Consented CIS patients complete similar questionnaires once per year, with data collected on episodes of inflammation, symptoms, treatments, investigations, contact with health professionals and whether the diagnosis of CIS has changed. Three validated questionnaires are also included (Table 1). Patients with CIS at baseline who are subsequently diagnosed with MS are switched to the six-monthly MS data collection schedule.

In addition to the patient-completed questionnaires, EDSS is assessed for patients attending neurology outpatient clinics at one of the neurology centres, but no specific clinic attendance is organised to evaluate relapse information. SWIMS is therefore very much patient-orientated, complemented by information from neurological appointments where available.

To examine the reliability of patient-reported MS subtype, we conducted a comparison of patient-reported MS subtype with neurologist evaluation using retrospective case note evaluation. All participants were asked in the baseline questionnaire to indicate which type of MS they considered that they had. Case notes for the first 396 participants by surname were reviewed by the project coordinator or neurologist for this purpose.

### Data management

Anonymised data are input to a bespoke Microsoft SQL Server 2005 database, using a double data entry system to eliminate data entry errors. Data are then exported to statistical packages including SPSS (version 15) and Minitab (version 15) for summarizing and statistical analysis.

### Data analysis

Data from 967 baseline questionnaires completed by participants with MS were collated and summarised using descriptive statistics to provide a comprehensive profile of this cohort.

## Results and Discussion

### Recruitment and follow-up

**Figure 1:**
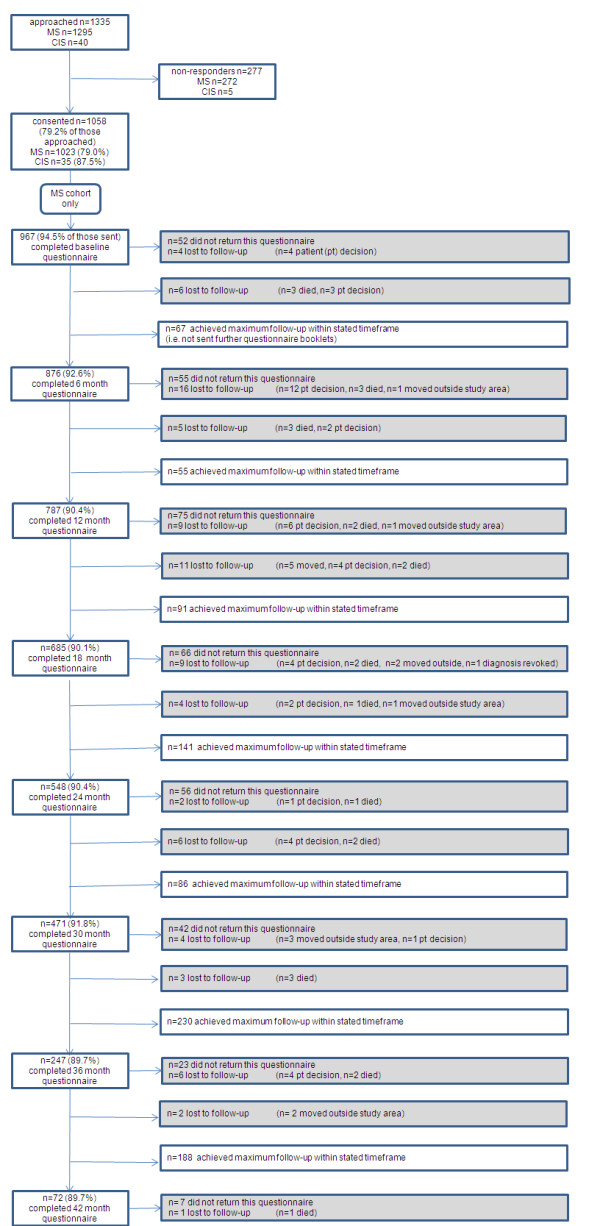
Disposition of individuals approached to participate between August 2004 and April 2008.

Figure 1 illustrates the disposition of MS patients approached to participate between August 2004 and April 2008 as well as follow-up rates during this time period. Within the same period, 40 invitations were sent to patients with CIS, of whom 35 (87.5%) consented to participate. Two participants with CIS were subsequently diagnosed with MS, and a further four participants with CIS were lost to follow-up (due to moving outside the study area).

Approximately 75% of those provided with information about the project between August 2004 and April 2008 consented to take part, which we consider to be a reasonable recruitment rate for this type of study. However, it is still important to understand why people chose not to take part. Reasons given to the neurologist for not taking part included a preference not to be reminded of the diagnosis of MS, particularly in the early stages of the condition.

The predominant source of recruits (63% of those recruited) was the consultant-led outpatient neurology clinic. A further 21% had been approached following review of the hospital notes, 10% were self-referrals, and 6% had been approached by their General Practitioner on behalf of the research team.

Median duration of follow-up is 2.0 years (n = 1023), with initial recruits to SWIMS having completed four years of follow-up. Eighty-eight people (8% of those consented) were lost to follow-up to the end of April 2008: 47 (4% of those consented) decided to withdraw, 25 (2%) died, 15 (1%) moved out of the study area, 1 (< 1%) had the diagnosis of MS revoked. The most common reasons for self-withdrawal were: "in poor health" and "questionnaires too difficult/burdensome". Questionnaire completion is consistently high, with 92% of the 5075 booklets sent to participants up to the end of April 2008 returned to the research team. The questionnaire return rates of around 90% are remarkable, and are being maintained by providing regular newsletters about the project and an appreciation by people with MS that the information being collected will lead to improved understanding and treatments in due course. The large sample size also provides reassurance of limited potential bias. Participant retention in long-term studies is a crucial issue when considering current methods of testing treatments in progressive MS. We are currently exploring methods of maximising retention to SWIMS, including use of internet data capture and on-line questionnaire completion.

Recruitment and data collection will continue for the foreseeable future, and we expect the information derived from this study to become increasingly useful over time. Because the project is a longitudinal study of patient-reported outcomes, we have excluded people with severe cognitive impairment, as we do not have validated methods for patient-reported data under these circumstances. This may inevitably lead to a degree of bias in the overall results in epidemiological terms, but this design will be able to address the aims of the project, namely to enable a better understanding of disease impact, to evaluate and improve on patient-reported outcomes, and to develop alternative prognostic models.

### Baseline data for MS patients

#### Participant demographics

Seventy five percent of the study sample (n = 967) was female, a ratio of 3.01:1.00. The age range at time of entry to SWIMS was 18.4 to 83.4 years, with a mean (standard deviation) of 51.6 (11.5) years (n = 961). Mean (standard deviation) age at disease onset, i.e. patient-reported date of first MS symptom, was 35.4 (10.8) years (n = 902). The median (inter-quartile range) time since first symptom of MS to time of entry to SWIMS was 13.3 (6.8 to 24.5) years (n = 934) and median (inter-quartile range) time since diagnosis was 9.9 (2.9 to 15.9) years (n = 921).

Diagnosis was reported to have been assisted using MRI in 84% of the sample. Thirty-five percent of the participants had MRI together with lumbar puncture and evoked potentials, whereas 8% of participants had been diagnosed on clinical grounds only. The demographics of the SWIMS sample were similar to other surveys of MS in regions of the UK, as reviewed by Fox *et al *[16].

#### Type of MS

Thirty six percent of participants reported that they had relapsing-remitting MS (RRMS) at time of entry to SWIMS, 21% had primary progressive MS (PPMS), 19% had secondary progressive (SPMS), 3% had a benign type and 21% did not know which type. When compared to neurologist assignment of MS subtype, agreement was achieved in 63% of the 396 cases reviewed. Cohen's kappa statistic was 0.48: a "moderate" level of agreement according to Altman[17]. The 95% confidence interval for kappa was 0.42 to 0.54.

The most common differences amongst the remaining cases were (a) when the participant was unsure of MS type and the neurologist was able to define subtype (n = 58, 15% of total), (b) where the participant felt they were in the RR phase, but the neurologist felt they were progressive (n = 31, 8%), (c) when there was disagreement between PPMS and SPMS (n = 28, 7%). and (d) when patients identified themselves as having progressive disease, but the neurologist had assigned RRMS (n = 12, 3%).

Defining MS subtypes can be an inexact science, even from the perspective of the neurologist. For example, in progressive disease, when an individual may present in middle age with a history of one or more possible neurological events earlier in life, it can be difficult to define subtype with certainty. Similarly, when an individual has had a diagnosis of RRMS and starts to acquire increasing disability, the precise definition of onset of secondary progression becomes blurred. Our experience is that patients often have difficulty in defining their own disease course, and the current levels of uncertainty are not unexpected.

#### Relapse data

Participants were asked to give details on any relapses that they had had in the previous twelve months. The number of relapses, by patient-reported MS type, is summarised in Table 2. Overall, 36% of participants were relapse-free in the previous twelve months. Across all participants, the mean (standard deviation) number of relapses was 0.9 (1.1) per year, whilst the mean (standard deviation) number of relapses in those reporting at least one relapse in the previous twelve months was 1.8 (1.0) per year.

**Table 2:** Patient-reported number of relapses in twelve months prior to baseline: percentage of participants experiencing relapse(s) by MS type

	Number of relapses in last twelve months					
**MS Type**	0	1	2	3	4	Don't know

Benign (n = 28)	54	39	0	0	0	7

Relapsing-Remitting (n = 345)	23	35	19	9	6	8

Primary Progressive (n = 182)	65	13	6	2	3	12

Secondary Progressive (n = 178)	33	34	9	7	3	14

Don't Know (n = 173)	30	23	15	7	3	22

All (n = 931)	36	28	13	7	4	13

Data for MS subtype or the number of relapses or both were missing for 36 participants.

The use of steroids in association with relapses, time off work due to relapses, hospitalisation and limitations in everyday activities associated with relapses are summarised in Table 3. Of all the 835 relapses in the previous twelve months, 157 (19%) resulted in no time off work, 61 (7%) resulted in less than one week off work, 38 (5%) resulted in one to two weeks off work and 106 (18%) resulted in more than two weeks away from work. For 394 relapse incidents (47%) the individual was not working. Data on work impact was missing from 79 relapses (10%). In total, 2% of relapses were treated with both oral and intravenous steroids, 12% with intravenous steroids only and 14% were treated with oral steroids only (at various doses).

**Table 3:** Patient-reported impact of relapse(s) in twelve months prior to baseline: percentage of relapses by relapse number

Relapse Number	Oral steroids	IV steroids	Time off work	Hospitalised	Limitations to everyday activities
1^st ^or only relapse (n = 477)	19	16	25	15	78

2^nd ^(n = 221)	14	11	24	7	76

3^rd ^(n = 99)	9	7	19	5	73

4^th ^(n = 38)	5	8	18	8	63

All relapses (n = 835)	16	13	24	11	76

Data on MS relapse can be notoriously difficult to interpret, with the presence of daily symptom variation and "pseudorelapse", in which a variety of mechanisms other than an increase in disease activity is responsible for symptom deterioration. Although many clinical trials have used the original Poser criterion [18] for relapse, of at least twenty-four hours of neurological deterioration, we opted for the more stringent definition of at least forty-eight hours of change in symptoms. This more stringent definition has previously been used in clinical trials (for example in Jacobs *et al*[19]) and it was felt to be more appropriate in the context of patient self-report, in order to minimise the potential for reporting of pseudorelapse.

Previous data on relapse frequency with retrospective assessment in cross sectional studies provide a figure of < 0.5/year, whereas prospective evaluation of relapses provides a higher figure, of between 0.5 and 1.0 relapse/year (reviewed in Confavreux and Compston[20]). Data from the present SWIMS cohort would therefore be consistent with other studies in which more formal evaluation of relapses has taken place, and suggests that patient report could be a valid and less resource intensive method for relapse assessment. Further work is needed to investigate this assumption and also to evaluate the impact of relapse on both overall disease course and from the patient perspective.

Not unexpectedly, some patients who classified themselves as either PPMS or SPMS continued to report having relapses, with the mean (standard deviation) relapse frequency being higher in the SPMS group (0.8 (1.1) per year) compared to the PPMS group (0.4 (0.9) per year). This is consistent with other data from PPMS populations; a significant minority (28% in one study[21]) of patients with PPMS reported an apparent relapse at some time during the course of the disease[22].

Although around three quarters of relapses were associated with limitation of everyday activities, only 11% of relapses resulted in hospital admission, which is consistent with an increasing tendency to use oral steroids rather than intravenous steroids. Not surprisingly, considerable time was lost off work due to relapse. Although previous studies have considered employment, e.g. Kobelt *et al *[23], very little work has been done on the full economic impact of relapse when adjusted for loss of earnings. The present SWIMS cohort sample is likely to have less bias than some previous work in this regard. For example, although Kobelt's study [23] included questionnaire data from over 13,000 people with MS, administered through the national MS Society, this represented a response rate of only 19%. The SWIMS data provide some insight into relapse impact in the era of disease modifying drugs, but much more work is needed to develop rigorous ways of evaluating relapse impact at all stages of the disease. The data would suggest that there is a need to provide a better definition of relapse from the patient perspective, without resorting to the EDSS or neurological examination. This may have implications for the use of disease modifying treatments, and further work investigating the relationship between patient-identified relapse and long-term disability would be useful.

#### Symptoms

Participants were asked to indicate their current symptoms, if any. Table 4 lists the percentages of all participants reporting symptoms at the time of completion of the baseline questionnaire, by patient-reported MS type.

**Table 4:** Symptoms reported at baseline: percentage of participants by self-reported MS type

	All (n = 967)	Benign (n = 29)	Relapsing-remitting (n = 347)	Primary progressive (n = 202)	Secondary progressive (n = 184)
Fatigue	80	38	80	83	83

Poor balance	75	35	64	90	89

Any pain, including visual pain	70	35	67	77	76

Any pain (not visual)	69	35	65	76	75

Muscle weakness	64	28	53	82	80

Problems with memory	57	35	58	57	59

Pins and needles/tingling	56	45	59	54	55

Decreased or worsening mobility	56	10	35	81	76

Muscle stiffness	54	21	48	64	67

Muscle spasms	53	17	44	66	67

Sensory loss/numbness	53	28	53	49	65

Loss of dexterity	52	21	41	65	70

Urinary urgency	49	38	44	49	57

Muscular pain	49	28	42	57	57

Problems with concentration	49	38	50	47	54

Urinary frequency	48	38	45	52	53

Constipation	44	28	40	48	52

Difficulties with co-ordination	43	14	33	56	56

Emotional lability	42	28	46	38	39

Joint pain	40	21	36	45	42

Feeling anxious	35	31	31	39	39

Urinary incontinence	30	24	19	39	41

Depression	30	21	28	31	30

Urinary hesitancy	29	14	24	31	39

Weight gain	28	10	23	33	32

Blurred vision	27	14	29	25	28

Sexual problems	27	14	23	29	36

Tremor	26	7	19	35	33

Burning pain	25	21	23	24	29

Shooting pain	23	10	21	23	26

Swallowing difficulties	21	7	13	27	28

Speech problems	21	7	16	26	25

Faecal incontinence	13	7	8	15	22

Other pain	13	10	12	13	14

Double vision	12	10	10	15	17

Weight loss	10	14	6	10	16

Diarrhoea	10	10	9	8	9

Painful vision	9	4	10	8	12

Colour desaturation	8	4	10	5	7

Pressure sores	4	0	2	8	6

A total of 205 participants did not know their MS type, or did not report an MS type.

It is not surprising that fatigue was the most commonly reported symptom across all participants. Regarding subtypes of MS, sensory symptoms were slightly more common than fatigue in the benign disease subgroup, and in both types of progressive disease (primary and secondary) balance difficulties were slightly more common than fatigue. Memory problems were reported by about 60% of patients, with the only major difference between subtypes being that benign disease was associated with less memory trouble (35%). Similarly, concentration difficulties were reported by around 50% of the participants, with few differences between subtypes except benign MS where the reported prevalence was 38%.

#### Medication

Participants were asked to indicate which medications they were currently taking, or had taken in the last twelve months. Of the total SWIMS participants (n = 967), 18.1% were currently taking or had previously taken some form of disease modifying therapy (including beta-interferon, glatiramer acetate, azathioprine, alemtuzemab, mitoxantrone and cyclophosphamide). Within the RRMS group 31% (n = 347) were currently receiving or had previously received disease modifying medication. The percentages currently on this type of medication were 23% of the RR group and 12% of the total sample. The commonest disease modifying drugs in use were one of the forms of beta-interferon and glatiramer acetate (22% of RR group and 11% of the total sample).

In order to obtain a picture of the possibly unmet need for symptom treatment in MS, we report symptoms and associated medication use in Table 5. Although 769 patients (80% of the cohort) experienced fatigue, only 3 people were taking amantadine or modafanil and were without fatigue. Fifty people (7%) were taking one of these medicines and continued to have fatigue. Among the large percentage of people with symptoms of bladder dysfunction, 26% were taking some associated oral medication. Five percent of people with bladder symptoms had obtained complete symptom relief, as indicated by the number of participants without current symptoms of bladder dysfunction but currently taking medication indicated for bladder dysfunction.

**Table 5:** Symptoms and associated medications use

Symptom	Number (%) of participants with current symptom	Associated medication	Number (%) of participants with current symptom		Number (%) of participants without current symptom
			**currently taking** **associated medication**	**not currently taking,** **but previously taken,** **associated medication**	**currently taking** **associated medication**

Fatigue	769 (80)	Amantadine	33 (4)	35 (5)	2 (1)

		Modafinil	17 (2)	16 (2)	1 (1)

Bladder	756 (78)	Desmopressin spray	6 (1)	9 (1)	0

		Desmopressin tablets	5 (1)	3 (0)	0

		Oxybutinin	93 (12)	64 (8)	6 (3)

		Tolterodine	63 (8)	25 (3)	2 (1)

		Trimethoprim	27 (4)	35 (5)	2 (1)

Pain (including visual pain)	681 (71)	Amitriptyline	123 (18)	85 (12)	19 (7)

		Carbemazepine	39 (6)	47 (7)	4 (1)

		Co-codamol	107 (16)	117 (17)	8 (3)

		Gabapentin	83 (12)	48 (7)	5 (2)

		Ibruprofen	127 (19)	194 (28)	28 (10)

		Nabilone	6 (1)	7 (1)	0

		Paracetamol	188 (28)	193 (28)	28 (10)

Spasticity	672 (70)	Baclofen pump	6 (1)	1 (0)	2 (1)

		Baclofen tablets	163 (24)	78 (12)	17 (6)

		Botulinum toxin	2 (< 1)	0 (0)	0

		Clonazepam	24 (4)	9 (1)	2 (1)

		Dantrolene	7 (1)	5 (1)	1 (< 1)

		Diazepam	26 (4)	64 (10)	4 (1)

		Tizanidine	45 (7)	16 (2)	2 (1)

Pain (excluding visual pain)	670 (69)	Amitriptyline	123 (18)	86 (13)	19 (6)

		Carbemazepine	39 (6)	47 (7)	4 (1)

		Co-codamol	104 (16)	117 (17)	11 (4)

		Gabapentin	82 (12)	48 (7)	6 (2)

		Ibruprofen	126 (19)	189 (28)	29 (10)

		Nabilone	6 (1)	6 (1)	0

		Paracetamol	185 (28)	192 (29)	31 (11)

Depression	484 (50)	Citalopram	34 (7)	15 (3)	3 (1)

		Fluoxetine	47 (10)	33 (7)	14 (3)

		Paroxetine	9 (2)	12 (3)	7 (2)

		Sertraline	13 (3)	3 (1)	3 (1)

Constipation	421 (44)	Fybogel	32 (8)	76 (18)	8 (2)

		Senna	65 (15)	98 (23)	7 (1)

Sexual problems	256 (27)	Viagra	34 (13)	16 (6)	9 (1)

Sexual problems (males only)	129 (54^1^)	Viagra	34 (26)	15 (12)	9 (1)

Tremor	248 (26)	Propranolol	10 (4)	7 (3)	5 (1)

^1 ^54% of males in study sample.

Over 70% of the SWIMS cohort experience pain of some type, most commonly treated with paracetamol or ibuprofen, both available without prescription in the UK. About 10% of the cohort who did not report having pain, continue to take such simple analgesics.

Symptoms of spasticity were also present in around 70% of the cohort, and about one third of people affected were taking some medication for this, with single figure percentages obtaining complete symptom relief (using the assumption given above for bladder dysfunction).

#### Contact with health and social care specialists

Participants were asked to indicate whether they had seen any specialists about their MS in the last twelve months and if so, how often (Table 6). Access to specialist services will vary both within and between counties. Asking about service contact, relapses and symptoms enables a broad picture to be created of the impact of MS on individuals and the ability of health services to deal with that impact. Considerable service demands are placed on primary care, even though each General Practitioner may only expect to have two to four patients with MS on their list. This is evident even when specialist nurses and neurologists with an interest in MS are available. At the other end of the scale, considering the prevalence of cognitive symptoms and pain, contact with clinical psychology and the pain management services is surprisingly low. It is difficult to know whether this reflects poor availability of services or problems in care pathways. Although there are some recommendations concerning the ratio of specialist nurses to population, there is comparatively little available data on case-mix for individual health specialists, making health planning difficult. Once again, there is a need for more work on the comparative success of treatments and support made available by each of the range of individuals that comprises the multidisciplinary team.

**Table 6:** Contact with specialists in twelve months prior to baseline: percentage of participants

	Not seen/ Not applicable	Once	2 to 4 times	≥ 5 times	Total number of responses
Chiropodist (or podiatrist)	86	4	6	4	811

Clinical Psychologist	97	1	1	< 1	795

Continence Advisor/Nurse	77	11	11	2	827

District Nurse	87	3	3	7	810

Dietician	96	2	2	< 1	799

GP	24	20	38	18	896

MS Specialist Nurse	54	25	19	2	857

Neurologist	28	44	26	1	904

Occupational Therapist	75	10	11	4	823

Ophthalmologist	78	17	5	1	825

Pain management service	96	2	2	1	804

Physiotherapist	54	10	19	17	851

Rehabilitation Doctor	97	2	1	< 1	803

Respite or Rehabilitation Unit (admitted for a period of respite care)	93	4	3	< 1	803

Social Worker	88	5	6	2	812

Speech Therapist	93	5	2	< 1	805

## Conclusions

Here we describe the rationale for SWIMS and report the baseline characteristics of a cohort of 967 people with MS. Overall, the data broadly reflect clinical experience in confirming high symptom prevalence with relatively little complete symptom relief. Establishing effective treatments for these symptoms must start with a full appreciation of all aspects of symptom impact.

SWIMS is enabling new scales to be developed (e.g. the MS Spasticity Scale, MSSS-88 [24]), and the performance of other scales to be evaluated (including the Fatigue Severity Scale [25], the General Health Questionnaire-30 [26], and the MS Walking Scale). Further qualitative work will be necessary in order to inform aspects of symptom impact useful for the creation of new measurement instruments.

## Competing interests

The authors declare that they have no competing interests.

## Authors' contributions

JZ conceived of the concept and design of the study. WI participated in the coordination of the study. JV and JH participated in the design of the study. SC performed the statistical analysis. DW participated in the design of the study and performed the statistical analysis. All authors participated in drafting the manuscript, and all authors read and approved the final manuscript.

## Pre-publication history

The pre-publication history for this paper can be accessed here:

http://www.biomedcentral.com/1471-2377/10/88/prepub
